# Simulation of turbulent effective wakes for propellers in off-design conditions by a correction factor approach

**DOI:** 10.1007/s00773-020-00794-7

**Published:** 2021-01-19

**Authors:** Antonio Sánchez-Caja, Jussi Martio, Ville M. Viitanen, Timo Siikonen

**Affiliations:** 1 VTT-Technical Research Center of Finland, Espoo, Finland; 2 Aalto University, Espoo, Finland

## Abstract

This paper presents a procedure for the estimation of propeller effective wakes in oblique flows. It shows how a recently developed method for controlling coupling errors can be applied to analyze propellers operating in off-design conditions. The approach allows the use of fast potential flow methods for the representation of the propeller in the context of viscous flow solvers and works accurately for a wide range of advance numbers and incidence angles with a minimum computational cost. The new method makes it possible to disclose flow phenomena on the effective wake that were hidden in conventional approaches of effective wake simulation. Different application cases are analyzed, such as a propeller-shaft configuration in inclined flow, a pod propulsor in an oblique inflow, and a ship hull advancing at a yaw angle. A dipole-like distortion on the effective wake is unmasked for a uniform flow incident to a propeller mounted on an inclined shaft. The flow component perpendicular to the axis is found to be responsible for the distortion. The effect of the direction of propeller rotation on the effective wake is illustrated for a single-shaft ship moving at a yaw angle. In particular, keel vortices are either attracted to or repelled from the propeller disk depending on the sign of the yaw angle or alternatively on that of the propeller rotation.

## Introduction

Traditionally, propeller designers have focused their design philosophy on the optimization of the propeller geometry for one ideal operational condition of the ship, e.g. straight motion for a given delivered power and ship speed. Recently, more holistic approaches in propeller design have been gradually adopted both for reducing operational costs at the various situations where the ship has to operate, and simultaneously, for improving energy efficiency and reducing noise and harmful gas emissions. In particular, international regulations concerning energy efficiency are being currently enforced [1] following a concrete implementation plan, which makes it more urgent for ship owners to rely on efficient propulsion systems and for propeller designers to develop efficient concepts.

One of the main obstacles that ship/propeller designers have to face in their ordinary work is finding the spatial and temporal distribution of the so-called effective wake at the propeller plane. Generally, the effective inflow at the propeller plane is derived from the analysis of the velocity field solutions obtained by coupling potential and viscous flow solvers. Sometimes, increasing the fidelity of the potential flow model for the representation of the propeller has not resulted in a more accurate prediction of hydrodynamic forces. Errors in coupling the flow solvers are one of the reasons for such unexpected behavior. They are especially relevant in oblique flows and high loadings. Frequently, contradictory claims about the benefits achievable with different types of propulsion solutions, (e.g. limits of energy saving with pre- and post- swirl devices) [2], are due to uncertainties in the effective wake prediction or to the use of incomplete information that relies only on nominal wake data. Errors in the prediction of the effective inflow are multiplied by a factor of two or three when evaluating propeller forces and moments [3]. Such errors are due e.g. to the difficulty in setting the location of the effective wake extraction on the propeller plane, or to potential flow models introducing jumps in flow quantities (e.g. in tangential velocities through the propeller plane or blade surfaces) in a way different than viscous flow models do, or to the spatial distribution of body forces, etc. They become significant especially in off-design situations, like those resulting in oblique inflows. In particular, maneuvering simulations need accurate flow inputs for reliable evaluation of hydrodynamic forces in ship trajectory predictions. Conventional numerical methods for the evaluation of effective wake are not accurate in such demanding situations. Similarly, propeller cavitation simulations require an accurate effective wake for the correct estimation of propeller cavitation behavior as pointed out, for example, in [4] for a propeller in behind condition in a straight course.

Several procedures for the estimation of the effective wake in the CFD context have been proposed over the years [5–7]. They combine potential flow methods for the simulation of the propeller action with RANS solvers for the simulation of the bulk flow around the hull. The propeller is usually modelled with a potential flow method based on either momentum theory, lifting line, lifting surface or panel methods. Propeller forces are modeled either as a pressure jump over the propeller disk or as body forces covering a 3D region extended over the RANS mesh, e.g. the volume swept by the blades over one revolution. Among recent literature, Rao et al. [8] and Guo et al. [9] coupled a panel method to a RANS solver, and the latter discussed various existing methods for estimating body forces. Huang et al. [10] pointed out the difficulties of estimating the effective wake in contra-rotating propeller (CRP) units. They coupled a vortex lattice method with a RANS solver using the same body forces in open water and self-propulsion simulations. Sun et al. [11] coupled a lifting line method to a RANS solver for self-propulsion simulation. The common feature of the current literature on effective wakes is that they are evaluated only for ship motions in a straight course.

A method for the estimation of propeller effective wakes in oblique flows has been recently developed [12], which addresses several sources of coupling errors. An interface in the form of an actuator disk works as coupling surface between the potential and viscous flow solvers. The location of the interface is the propeller plane, which avoids the need for extrapolations from upstream planes or surfaces. There is no restriction on the type of potential flow models that can be used (e.g. lifting line, panel method, etc.). The solution of the propeller solver is expressed in terms of an equivalent actuator-disk solution and then is transferred to the interface.

The method is an extension to inclined flows of an approach based on correction factors previously developed for the estimation of effective wakes in *straight* course [3]. The approach converts propeller-induced velocities approximately predicted via potential flow theory into ‘viscous’-induced velocities by means of a viscous flow RANS analysis. The correction factors are a function of both the radial and angular coordinates on the propeller disk. They are calculated for one reference advance number and work accurately in a neighboring, continuous region of advance numbers and inflow directions. This procedure permits calculation of the effective wake more precisely in off-design situations, reducing the CPU time, and therefore, enlarging its range of applicability to situations like those resulting from ship maneuvering. The method has been applied in [13] to the evaluation of the effective wakes in straight flow for a set of three CRP propellers. The reference shows the theoretical limits for the optimization of co-axial multi-propeller units.

This paper presents first a short description of the numerical method for the effective wake prediction. Extensions of the method are proposed to cases in which the direction of the effective inflow is not known a priori, and flow-direction updating is required together with correction factor updating during the iteration process of the viscous-flow solver. The robustness of the procedure is shown for an idealized case of propeller-hull interaction where the correction factors are evaluated once for an inclined flow situation, and they work accurately for other yawed and straight course conditions. Validation simulations are presented with selected propulsors in oblique flow for both a single propeller/shaft configuration in inclined flow and a podded propulsor subject to a yawed inflow. The influence of the correction factors is shown for such cases and for a ship hull of a single shaft propeller moving at a yaw angle.

## Numerical method

**Fig. 1:**
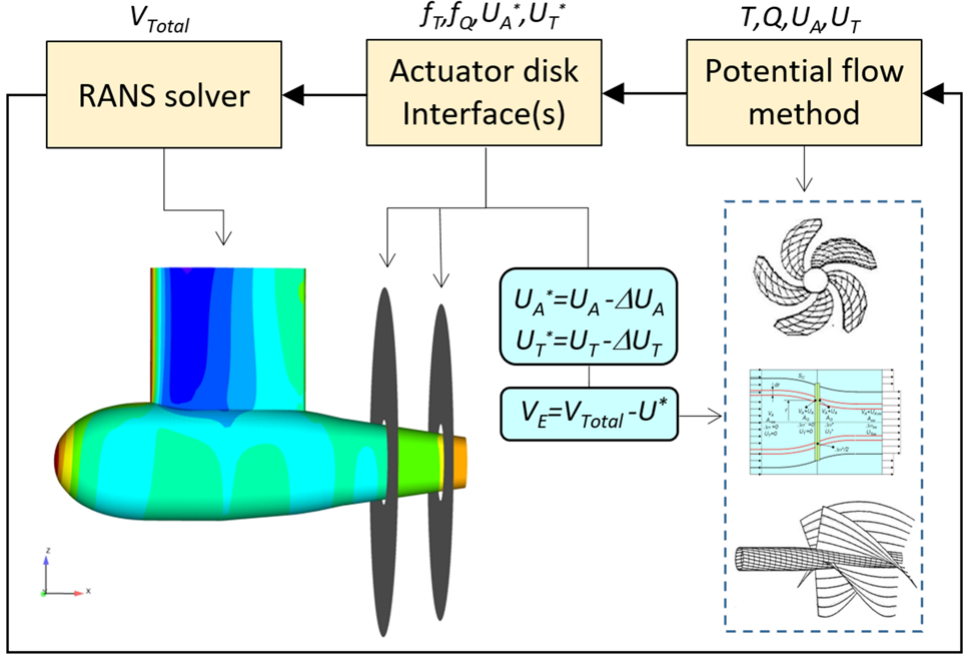
Notional scheme for the evaluation of the effective wake on a generic CRP/tandem pod unit

The viscous flow simulation is based on the solution of the RANS equations by either the artificial compressibility or the pressure correction method using RANS solver FINFLO. The viscous solution is extended to the wall, using cell sizes on the solid boundaries so that *y*+  is around one. A detailed description of the numerical method including discretization of the governing equations, solution algorithm, boundary conditions, etc. can be found in [14, 15]. The conceptual approach for the estimation of the effective wake is explained in [3, 12, 16]. A correction factor scheme is employed for the cancellation of the numerical errors derived from coupling a potential flow method with a RANS method (Fig. 1).

First, the geometries of one or several propellers integrating the propulsor unit are analyzed by a potential flow method, either lifting line, momentum theory, lifting surface or panel method. Next, the calculated propeller forces and moments (*T, Q*) are expressed in terms of body forces (*f*_T_*, f*_Q_) over an actuator disk interface. The body forces are a function of the radius and angular location over the disk (spatially varying), and time-independent in static analyses. The interface is inserted into the viscous flow solver (RANS) either as a part of the computational grid or as an external Chimera block. The interface thickness may consist of only one or more layers of axial cells at the location of the propeller plane where the body forces are distributed. The RANS solution provides a new effective inflow (*V*_E_) at the location of the propellers after subtracting the local overall propeller-induced velocity (U^*^) from the total velocities of the bulk flow (*V*_Total_). The propeller induced axial and tangential velocities (*U*_A_*, U*_T_) are corrected from coupling errors before subtraction (*U*_A_^*^, *U*_T_^*^). The procedure is repeated either for each RANS iteration when a fast propeller model (e.g. lifting line) is used, or after a fixed number of iterations in other cases (e.g. panel method).

## Correction factors

For an idealized uniform inflow *V* at a yaw angle α, incident on a propeller in the presence of no other surrounding bodies, the axial effective wake is *V*cos α,

1 $$\frac{{{V_{A,{\text{Bulk}}}} - U_{\text{A}}^{{\text{exact}}}}}{V} = \cos \alpha$$

where *V*_A,Bulk_ is the total axial velocity of the bulk flow in the RANS solver at the propeller plane and *U*_A_^exact^ is the exact propeller-induced axial velocity within the RANS context.

However, the induced axial velocity predicted by the potential flow solver *U*_*A*_ will differ from the exact one and a correction to *U*_*A*_ can be expressed as follows,

2 $$\frac{{\Delta {U_A}}}{V} = \cos \alpha - \frac{{{V_{A,{\text{Bulk}}}} - {U_A}}}{V}$$

where ∆*U*_*A*_ represents the correction term to be applied so that the potential-flow induced velocities *U*_*A*_ could be converted into ‘viscous’-flow induced velocities, *U*_*A*_ − ∆*U*_*A*_.

Axial correction factors (*F*_*A*_) that are independent of the advance number (e.g. *J*_*0*_, *J*_*1*_) can be defined,

3 $${F_{A0}}\, = \,\frac{{{U_{A0}}\, - \,\Delta {U_{A0}}}}{{{U_{A0}}}}\, = \,\frac{{{U_{A1}} -\Delta U_{A1}}}{{{U_{A1}}}}\, = \,{F_{A1}}$$

which allows calculation of the correction terms for other propeller loadings. They eliminate in an ‘exact’ way errors that depend linearly on the propeller induced velocities, and in an approximate way those that depend nonlinearly. For the justification of the approach and for treatment of the tangential and radial induced velocities and correction factors see [3, 12].

**Fig. 2:**
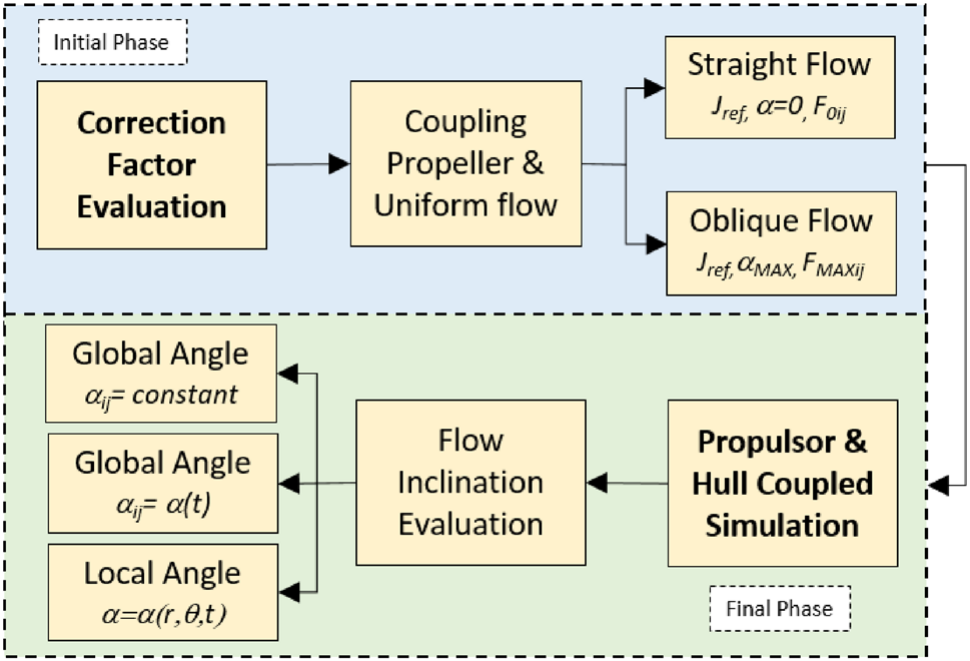
CF evaluation scheme for an ideal isolated propeller in uniform flow (Initial Phase), and CF implementation scheme for an actual coupled propulsor-hull simulation (Final Phase)

Figure 2 summarizes the implementation of the correction factor (CF) technique within the RANS-potential flow coupling approach. First (*Initial phase*), the correction factors are calculated at different radial and angular locations (*r,θ*) on the propeller disk for the idealized situation of an isolated propeller in uniform flow at a reference advance number (*J*_ref_). They are evaluated both for straight inflow (*F*_0_) and for an oblique inflow around the maximum expected angle in the actual simulation (*F*_MAX_). Next (*Final phase*), the correction factors for arbitrary angles in the final hull-propulsor coupled simulation are found by interpolation. Due to the symmetry of the solution, the correction factors for generic oblique angles can be obtained from at least a three-point interpolation between *α*, 0 and −*α*, where the negative angle solution is derived from the positive one by an 180° rotation shift of the correction factors.

In principle, the CF distribution may be selected from an oblique angle corresponding to the global expected average inflow over the propeller disk. Such global angle can be easily guessed in propulsion units like azimuthing pulling podded propulsors in uniform conditions. However, in most cases, i.e. conventional propellers located behind a ship hull operating at off-design conditions (e.g. ship yawed motion during manoeuvres), the direction of the effective inflow to the propeller is not known beforehand, and an iteration procedure must be followed to estimate it. The procedure can be implemented within the RANS iteration loop. We will illustrate in Sect. 3 the flow-rectifying effect of the ship hull in yaw motion, which modifies strongly the actual effective flow direction far beyond what is expected from the ship yaw angle.

**Fig. 3:**
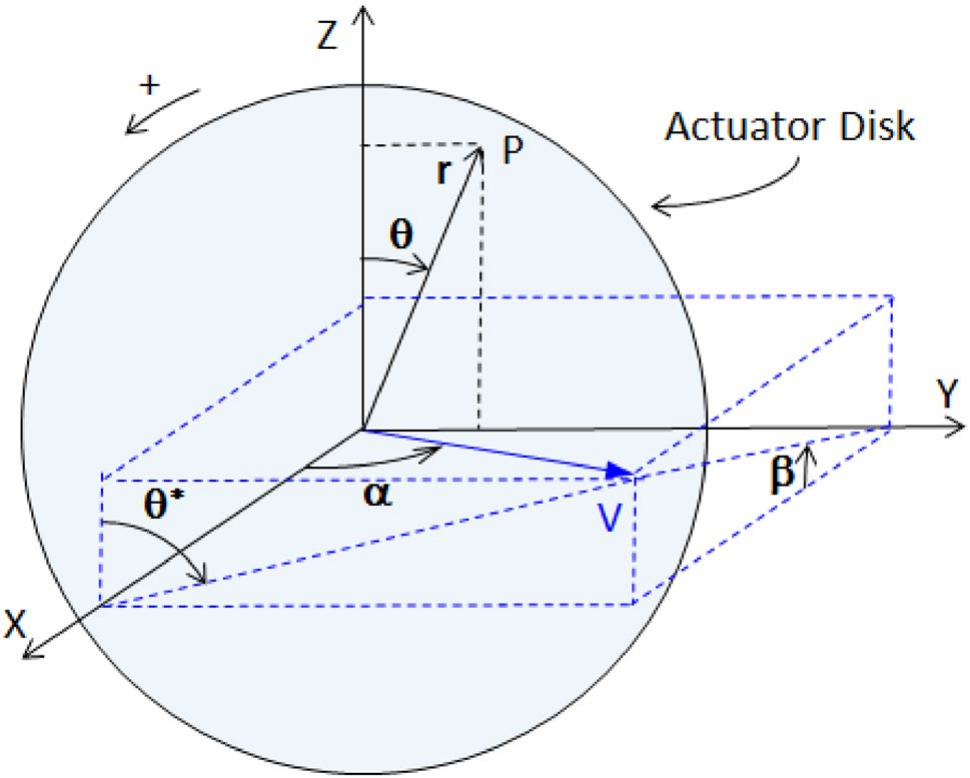
Definition of parameters related to correction factors. For θ* = 90°, *α* is the yaw angle. For θ* = 0°, *α* is the vertical inclination angle

The local direction of the flow is defined by two angles, *α* and θ*. The former is the angle of the velocity vector relative to the propeller axis, as described in Fig. 3. The latter is the angle between the vertical plane OXZ and that containing both the velocity vector and the propeller axis.

For cases in which the direction of the flow is not known beforehand, global angles varying in time can be defined as

$$\alpha {\mkern 1mu} = {\mkern 1mu} \cos ^{{ - 1}} \frac{{\iint {V_{x} }{\text{d}}r{\text{d}}\theta }}{{\left\| {\iint {\left( {V_{x} \vec{u}_{x} {\mkern 1mu} + {\mkern 1mu} V_{y} \vec{u}_{y} {\mkern 1mu} + {\mkern 1mu} V_{z} \vec{u}_{z} } \right)}{\text{d}}r{\text{d}}\theta } \right\|}}$$

4 $$\theta ^{*} {\mkern 1mu} = {\mkern 1mu} \cos ^{{ - 1}} \frac{{\iint {V_{z} }{\text{d}}r{\text{d}}\theta }}{{\left\| {\iint {\left( {V_{y} \vec{u}_{y} {\mkern 1mu} + {\mkern 1mu} V_{z} \vec{u}_{z} } \right)}{\text{d}}r{\text{d}}\theta } \right\|}}$$

where the $\overrightarrow{u}$ vectors are unit vectors along the axis directions, being OX the direction of the propeller shaft. The local velocity vector $\overrightarrow{V}$ is represented by its Cartesian components along the axes. The surface integrals are extended over the propeller disk. A further refinement of the method would be using the local effective inflow directions at different radial and angular positions as the basis for CF interpolation.

Summarizing, depending on the case at hand, we can consider three possible ways of estimating the direction of the inflow to the propeller, which corresponds to three possible directions,

(i) a *nominal* direction that is not evaluated but estimated from the particular conditions of the physical scenario under investigation; such direction may be constant in time and is constant in space over the disk,
(ii) a *global effective* direction that can be evaluated as an average value over the propeller disk; such direction may be variable in time and is constant in space, and
(iii) a *local effective* direction that can be evaluated as a local value over the propeller disk at each angular and radial coordinate; such direction may be variable in time and space over the disk.

From a practical point of view, the two first ways of estimating the direction are simple and efficient. They are employed in the application cases shown later in the paper. Notice that in all cases we are using the local velocities over the disk for the calculation of the local body forces and effective wake.

## Application cases

Next, some examples illustrate the application of the method in particular cases, showing the impact of the coupling errors.

### Propeller in inclined flow

Boswell et al. [17] conducted experiments with the 4661 propeller in a towing tank with inclined inflows up to 30°. They measured the periodic single-blade loads and compared them to results from available potential flow models. The models systematically underpredicted the experimental values of the unsteady blade loads, from about 20 percent at design condition to larger figures at off-design.

Recently, Martin et al. [18] repeated the viscous flow simulation using either the actual discretized geometry of the propeller or a lifting surface representation of the blades coupled with the viscous solver. The hub geometry was included. The average error at a range of advance numbers was about 11 percent for the discretized geometry and 25 percent for the coupled approach. The blade load non-dimensionalization was made somewhat differently from that in Boswell paper since the carriage velocity was employed as reference speed instead of the projection on the propeller axis.

**Fig. 4:**
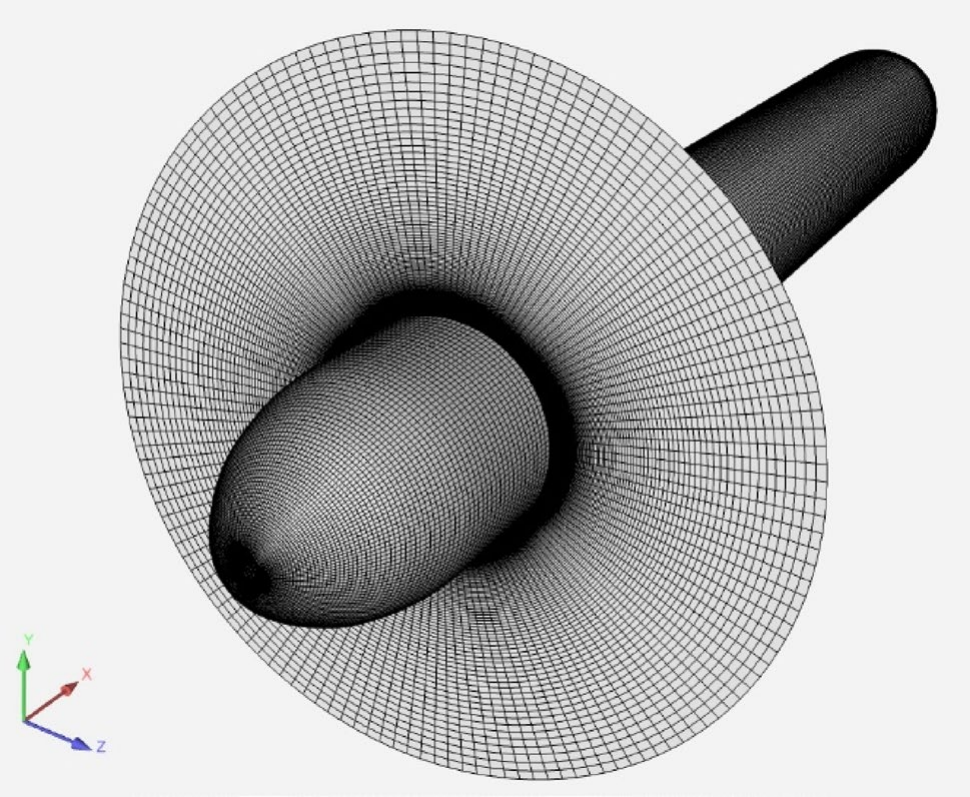
Computational grid for the 4661 propeller simulation using a coupled approach. Actuator disk interface

We have repeated the coupled computation using the CF approach. A structured mesh of 10 million cells was used for modelling the propeller hub. The grid is shown in Fig. 4 on the hub and at the propeller location. The potential-flow solver for the propeller was a quasi-steady lifting line code. The simplicity of the solver was chosen to illustrate the ability of the method in controlling different errors present in the simulation.

The lifting line method was first tuned in straight flow. The open water curve of the propeller in straight flow was reproduced with enough accuracy using standard lifting surface corrections [19] and a drag coefficient of 0.010. The differences from experiments were around one percent for both thrust and torque coefficients at the evaluation point. The correction factors were then calculated at the angles of inclination for a hubless geometry.

**Fig. 5:**
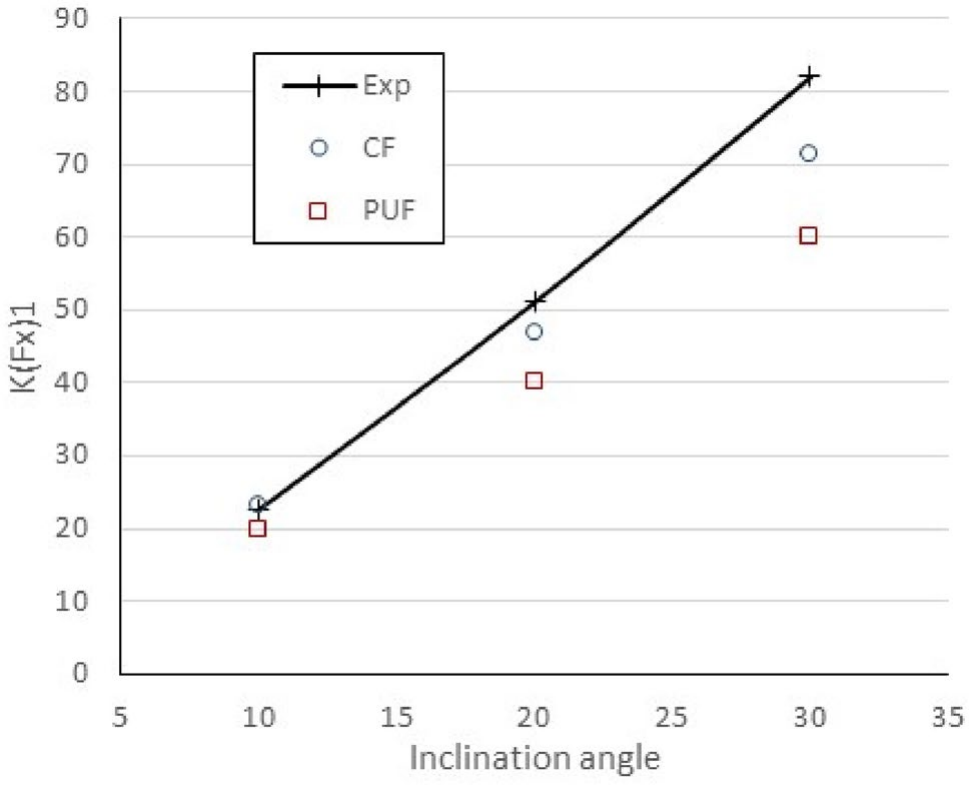
Comparison of first harmonic amplitude of propeller forces calculated with the correction factor (CF) approach versus experiments (Exp.) and results from [17] (PUF). *J* = 1.14

The RANS equations coupled to the potential flow code were solved for the inclined shaft mesh with the actuator disk interface. Figure 5 shows the non-dimensional first-harmonic amplitude for the axial force calculated with the correction factors compared to experiments at different inclination angles. *K(Fx)*_*1*_ is *K*_*T*_*/J* as defined in [18], i.e. using the reference carriage speed. Results from a lifting surface method (PUF) with uncorrected conventional coupling are also presented.

**Fig. 6:**
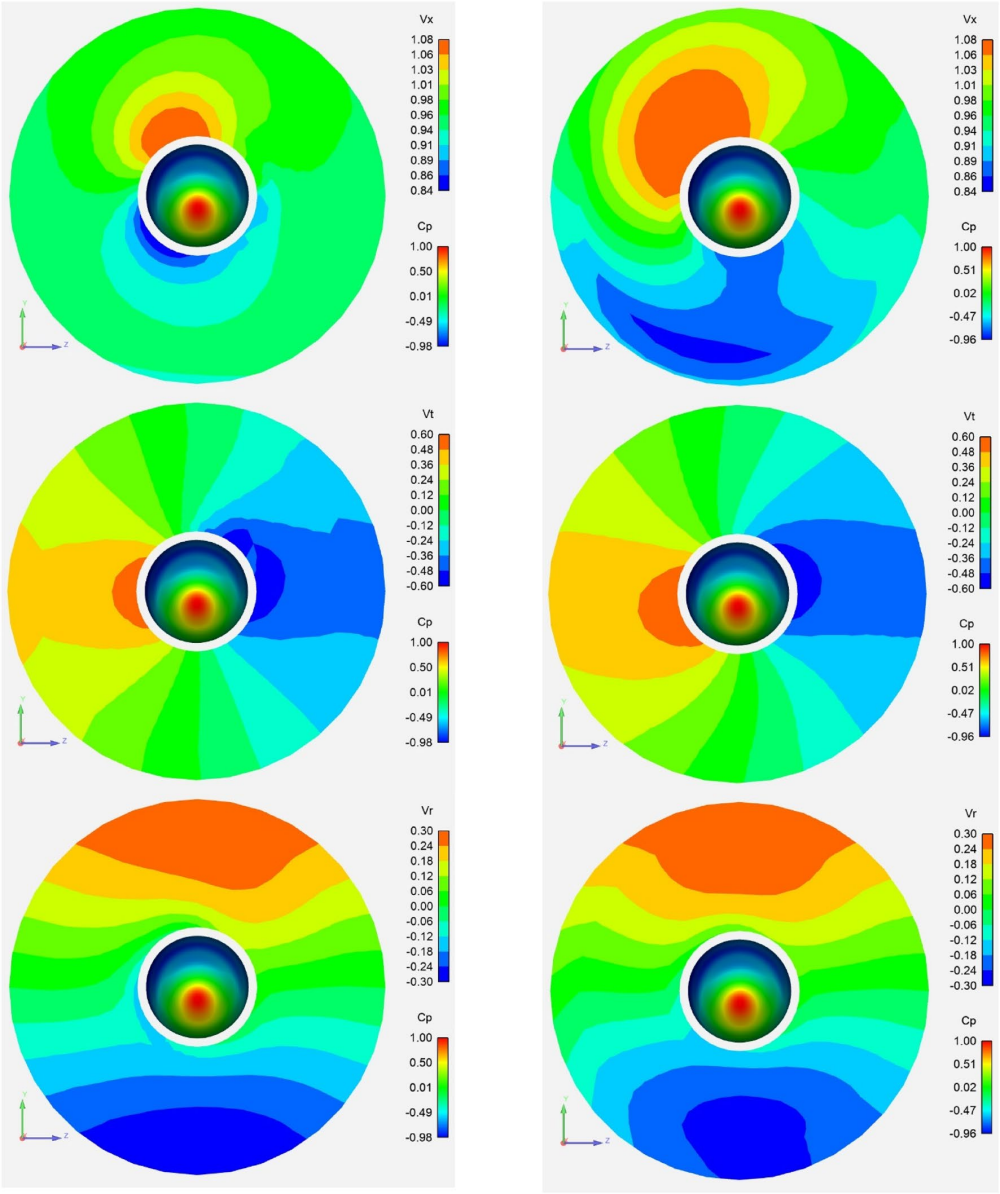
Axial (upper), tangential (middle) and radial (lower) effective wake velocities for 20° inclination angle and upward flow. Computations with (left) and without (right) correction factors. The hub is coloured by pressure. View from upstream. The velocity is made non-dimensional with the inflow at infinity

Figure 6 shows the axial, tangential and radial components of the effective wake on the propeller plane for an upward flow inclination angle of 20° using a discrete colour frame. The visible zone covers 110 percent of the propeller radius. The pressure field on the hub is shown with a continuous colour frame. The stagnation zone on the hub is coloured in red. The computations were made with (left side) and without (right side) correction factors.

It is interesting to notice that the highest and lowest values of the effective axial velocities are not located on the upper and lower sides of the hub as it would be expected for a vertically inclined inflow with a dominant axial component. They both are shifted circumferentially to the left as it would happen to the stagnation points of an upward flow over a cylinder with a counter-clockwise vortex on the cylinder axis. In fact, the propeller is rotating counter-clockwise when looking downstream and a circumferential flow is induced similar to that of an axial vortex. The scalar axial component of the flow is shifted accordingly. For the computation without correction factors, such effect is not clearly visible in the axial effective wake, since it is concealed by coupling errors spread over the disk. In the effective tangential velocities, the effect appears as an expansion of the downward tangential velocity peak close to the hub (right side in the figure) and a contraction of the upwards velocity peak on the left side.

### Pod propulsor

**Fig. 7:**
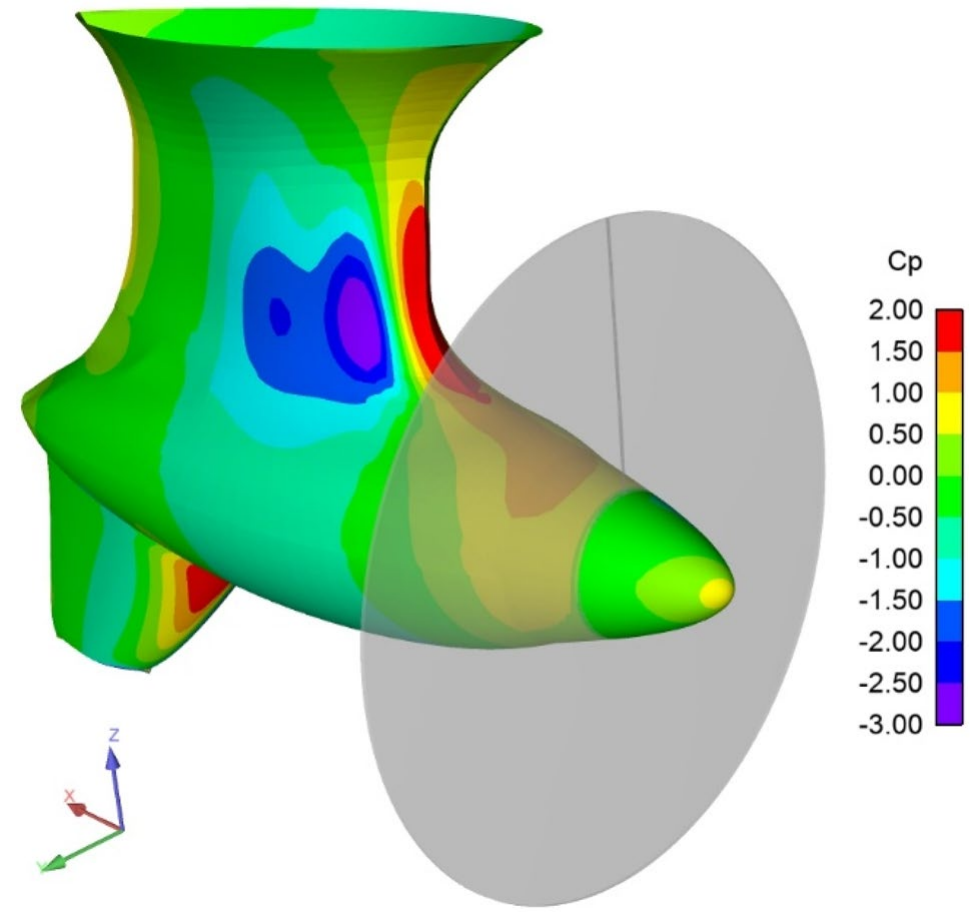
Podded tractor unit modelled with actuator disk interface. Pressure distributions on housing. Oblique flow case. Yaw = −8° and *J* = 0.65

A pulling podded propulsor unit is analyzed in this section and the numerical results are compared to model scale tests. The podded propulsor consists of a strut, a tail fin and a pod housing. Figure 7 shows the unit with the pressure contours for a −8° yaw angle and an advance number of 0.65. The pressure coefficient is made non-dimensional using as reference speed the inflow at infinity upstream. The main data of the propeller is given in Table 1.

**Table 1:** Propeller main characteristics

Propeller	
Diameter, *D* (m)	0.192
Pitch diameter ratio, *P/D*	1.12
Expanded area ratio, *A*_E_*/A*_0_	0.55
Hub radius ratio, *R*_hub_*/R*	0.25
Blade number	4

The propeller rotates at 12 rps. The flow is in the positive OX direction. Grids of 0.6 and 4.8 million cells were built yielding differences in force coefficients smaller than 0.5%, which is indicative of small numerical uncertainty. The fine mesh used in the final computations had O topology around the strut with 240 cells around the strut profile, C-topology around the lower half part of the pod with 72 cells in the circumferential direction, and C-topology was used around the lower fin with 128 cells around the profile. The grid in the actuator disk zone had 144 cells in the circumferential direction and 72 cells in the radial direction. The SST k-ω turbulence model is used in the simulations [20].

The lifting line code with lifting surface corrections was able to simulate accurately the performance of the single propeller in open water (straight flow) especially for advance numbers around 0.6–0.7.

**Fig. 8:**
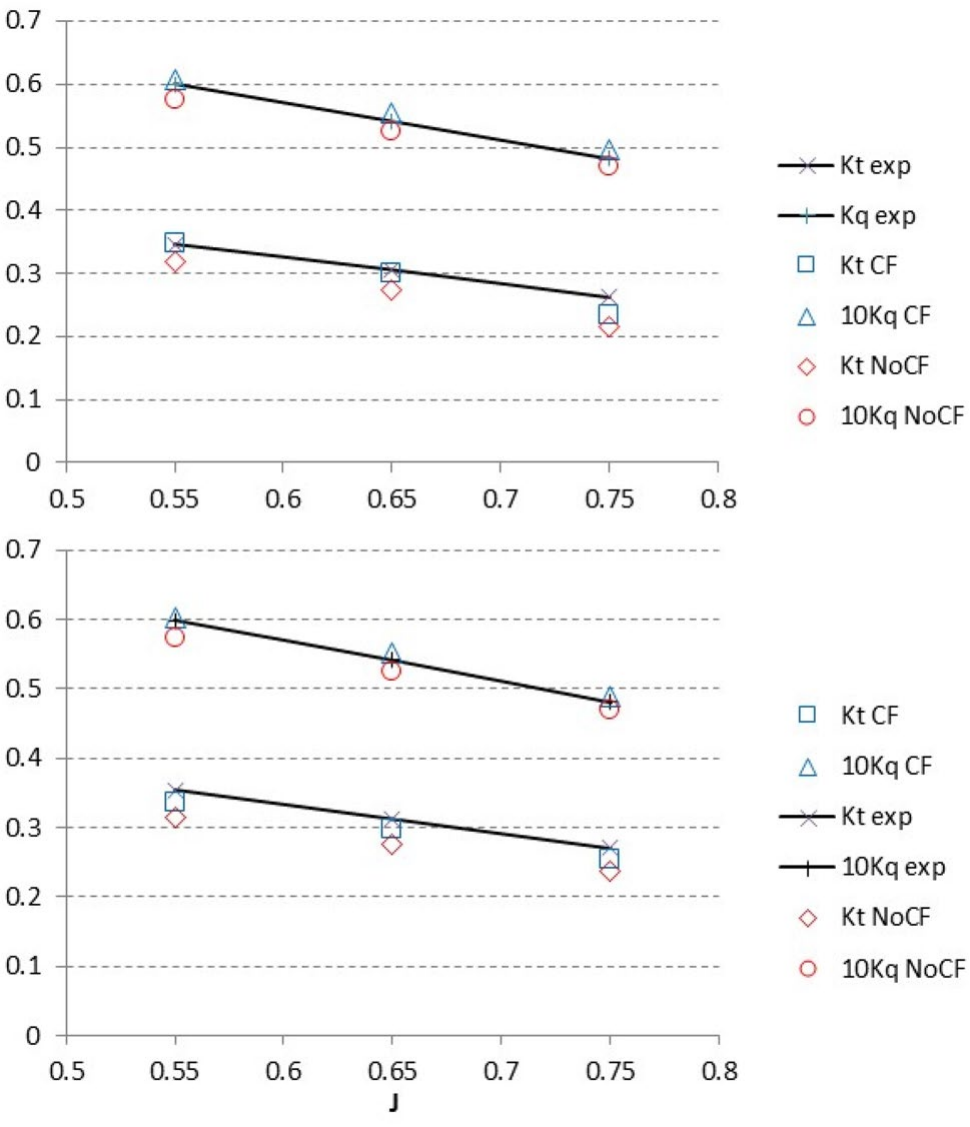
Performance coefficients for a podded unit at model scale. The inflow is at a yaw angle of 8° (up) and −8° (down). Corrected (CF) and non-corrected (NoCF) results are compared with experiments

Figure 8 shows the effect of the CF approach on the performance coefficients of the pod unit at  ± 8° yaw angle. The experiments were standard static open water tests conducted in a model basin. Coupling errors were reduced to 8% and 5% in *K*_T_ and *K*_Q_ respectively, for this application with an impact on the efficiency of 3%. After corrections, the fully turbulent flow regime in the computations seems to be the reason for the residual underestimation of the unit thrust relative to experiments where the flow is expected to be partially laminar.

**Fig. 9:**
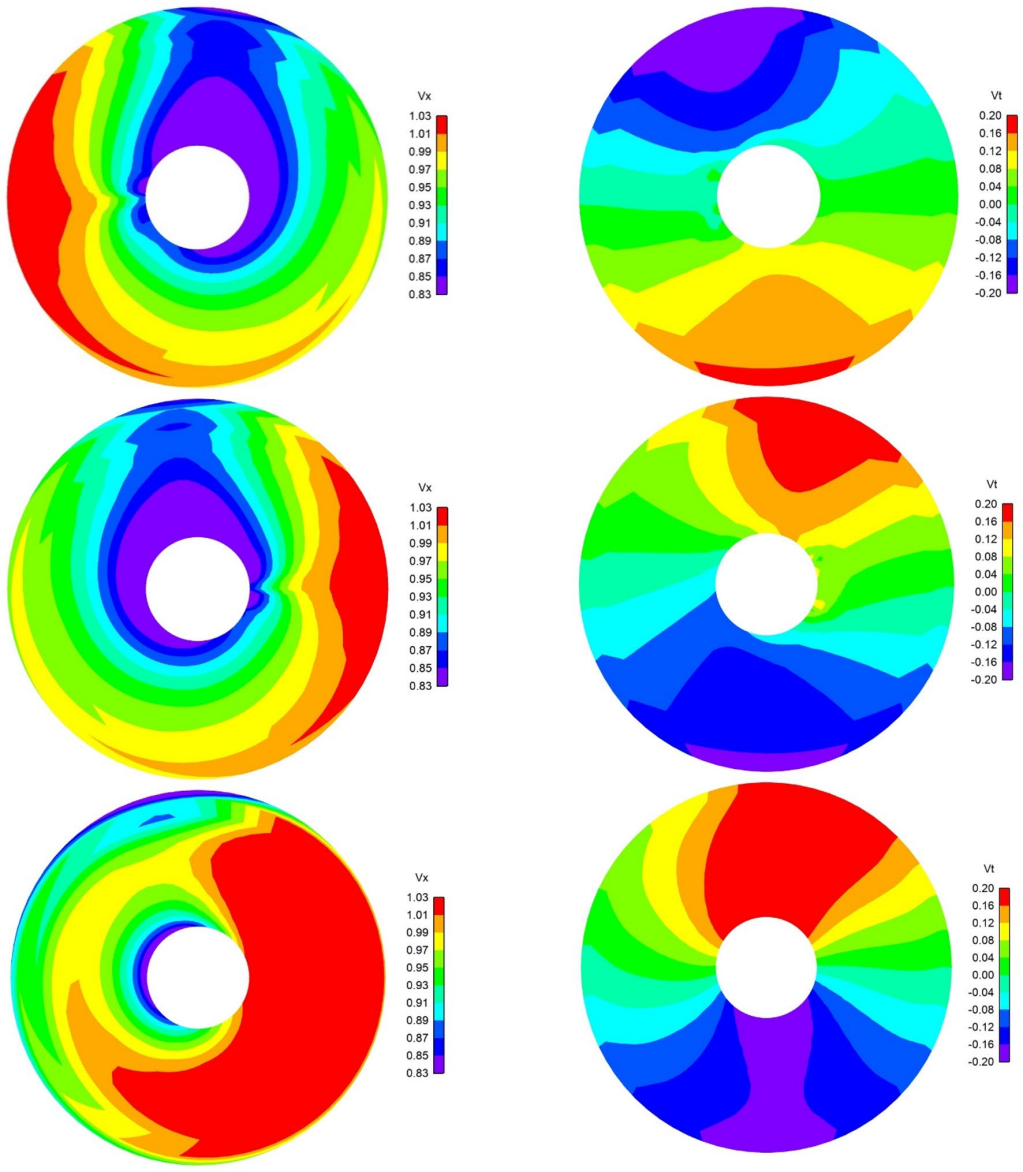
Axial (left) and tangential (right) distribution of non-dimensional effective wake velocities at *J* = 0.65 for 8° (upper) and −8° (middle, lower) yaw angles. The lower pictures are without the correction factors. The turning direction of the propeller (right-hand-side, counter-clockwise) used in the computations makes the results not to be fully symmetric. View from upstream

Figures 9 show the effective wake for  ± 8°, respectively, at *J* = 0.65. The velocity is made non-dimensional with the inflow at infinity. The right hand-sided propeller used in the computations (propeller rotating counter-clockwise in the figure) makes the results not to be fully symmetric, which is captured by the numerical approach. The figure is seen from upstream, and the positive yaw angle is ‘flow advancing towards the left’. The positive sign for the tangential velocity is counter-clockwise looking downstream, and for the radial velocity, outward. The visible zone is for radial stations, *r/R*$\in$ [0.30, 1.10].

Low axial velocities are visible slightly rotated clockwise/counter-clockwise in front of the strut because of the positive/negative yawed inflow, respectively. The low-velocity peak is somewhat wider for positive yaw, where the direction of the propeller induced flow and that of yaw add together. Similarly, a counter-clockwise angular shift is shown for the negative peak of the tangential velocity due to the interaction of the strut and yawed inflow. The thin fin located far downstream affects the effective field to a lower extent. The positive slope of the hub shape enforces positive radial velocities at the lower radial stations. The computations without corrections are also visible.

### Hull flow on a single shaft conventional propeller

Finally, the effective wake due to the interaction of a hull and a propeller is estimated. The main particulars of the hull are shown in Table 2. A double model boundary condition was enforced on the free surface. The reference four-bladed propeller had a 0.97 pitch-diameter ratio and a 0.6 expanded area ratio. A structured grid of 2.2 million cells was used in the computations. The mesh of the hull had O–O topology with 240 cells around the waterline of the hull, 64 cells in the direction of the frame line, and 128 cells in the direction perpendicular to the hull with hyperbolic tangent stretching and y + around 1. The mesh included an additional block containing the actuator disk of about 0.2 million cells. The SST k-ω turbulence model was used in the computations. The yaw angles for the inflow were −10°, 0° and 10°.

**Table 2:** Ship main particulars

*L*_pp_	67.0 m
*L*_overall_	77.1 m
*B*	12.0 m
*T*	3.6 m
Scale	1:12

The influence of the correction factors on the hull drag coefficient in the ship longitudinal direction (*C*_X_) was around 1% as shown in Table 3. However, the impact on the propeller performance coefficients (*K*_T_, *K*_Q_) reached up to 10% in thrust coefficient and 8 percent in torque coefficient.

**Table 3:** Influence of correction factors on propeller and ship performance coefficients

Yaw	% *K*_T_	% *K*_Q_	% *C*_X_
0	8.6	6.6	0.9
10	10.4	7.7	1.2
−10	9.2	6.9	0.6

**Fig. 10:**
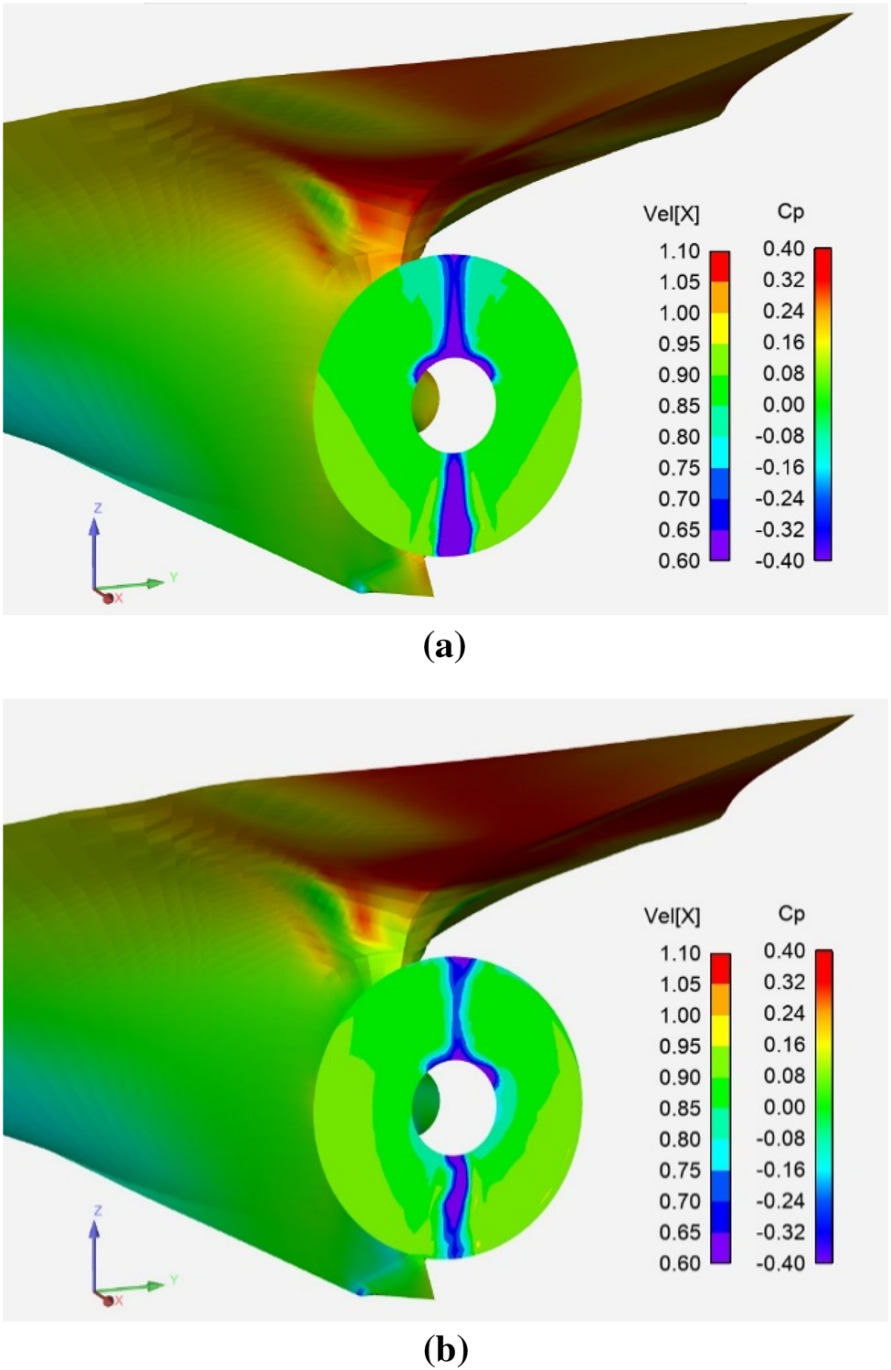
Nominal (**a**) and effective (**b**) axial wake for a 0° yaw angle. Pressures are visible on the hull

Figure 10 shows the axial nominal and effective wake for a non-yawed inflow. The effect of the propeller suction is to reduce the boundary layer thickness and low-velocity peaks in the effective wake. Pressures (*C*_*P*_) are visible on the hull. The pressure coefficient is again made non-dimensional using as reference speed the inflow at infinity upstream.

**Fig. 11:**
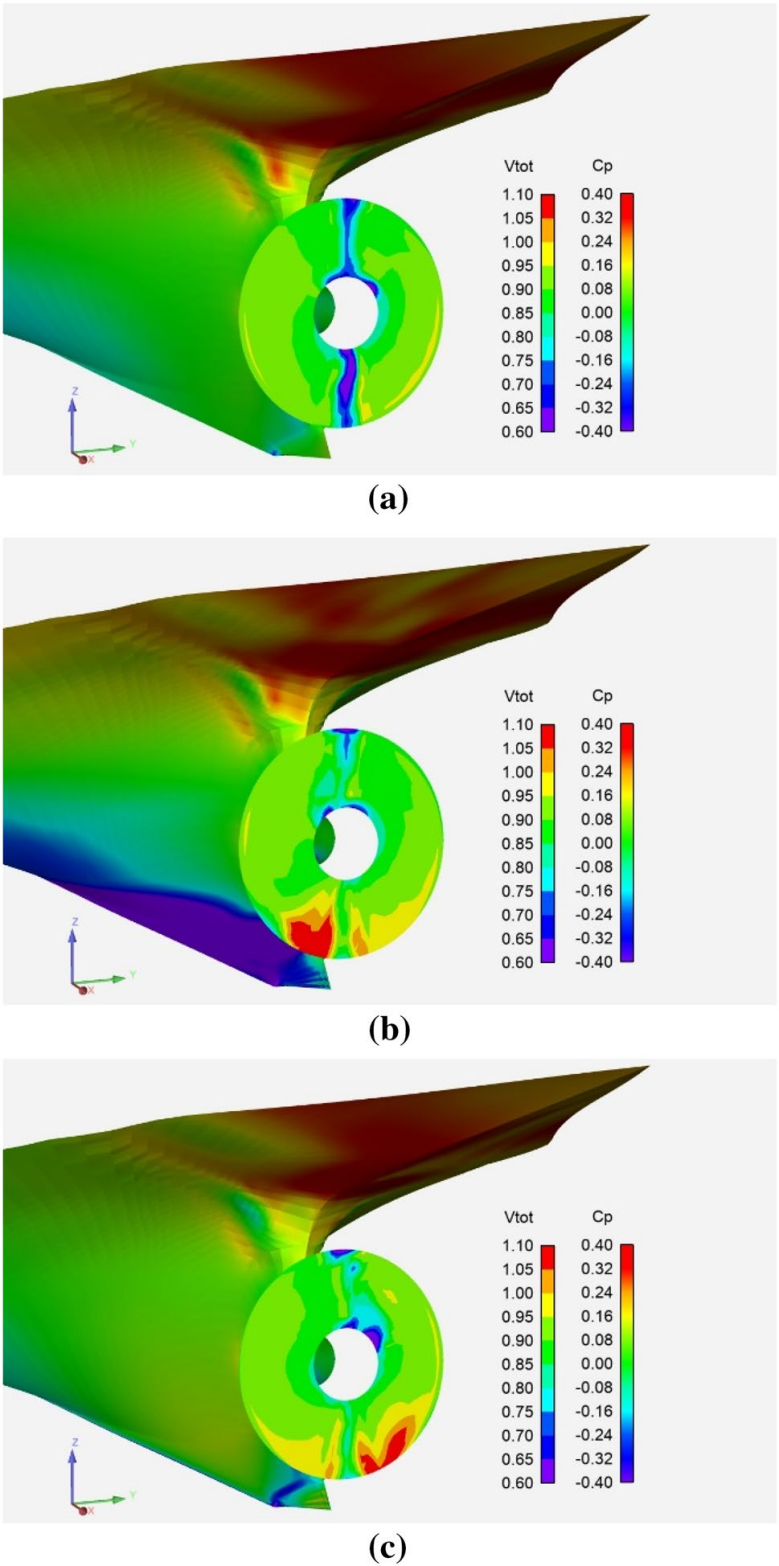
Total effective wake on the propeller plane for 0° (**a**), −10° (**b**) and 10° (**c**) yaw angles

Figure 11 shows the *total* velocity effective wake for different yaw angles. The sharp edges of the hull at the stern below the level of the propeller axis develop two counter-rotating vertical-axis vortices, which are visible for 0-yaw angle as a low-velocity blue line at the symmetry plane surrounded by larger velocities green/yelow areas on both sides. The RHS turning propeller encounters larger angles of attack on the upper starboard side due to the tangential flow induced by the hull flow. Consequently, larger local propeller loadings induced in turn larger velocities affecting the effective wake in that zone.

For the −10° yaw angle, the rotation of the propeller induces an up-flow on the port side, which results in the keel vortex (red zone on port side) entering the propeller disk and interacting with the vertical axis vortices. Conversely, for the 10° yaw angle, the downward flow induced by the propeller on the starboard side shifts the keel vortex away from the propeller disk (red zone on starboard side only visible at the disk edge at 5–6 o’clock positions).

### Global effective flow direction

Table 4 compares the directions of the flow at infinity upstream with those of the average effective flow at the propeller plane for the three cases analysed in the previous sections. For an extreme inclined shaft case of 30°, the shaft produces a disturbance lower than 2° in the incidence angles, i.e. no significant error is introduced by using a constant incidence angle equal to that at infinity for the evaluation of the correction factors. The situation is similar for the pulling pod case, even though the inclination angle of the plane containing the propeller axis and the flow direction changes about 5°. By contrast, in the third case, the hull rectifies strongly the flow both reducing its angle to the propeller axis and altering the position of the inclination plane. The impact of the flow direction used for the evaluation of the correction factors on the overall thrust and torque coefficients is also shown in the table. The larger impact is for the hull-shaft combination, which in any case is smaller than 1.5%.

**Table 4:** Variation of the averaged inclination angles of flow at the propeller plane (propeller) relative to inflow upstream (infinity)

	Inclined shaft	Pulling pod	Hull–shaft combination
α_infinity_	30.0^o^	8.0^o^	10.0^o^
α_propeller_	28.7^o^	9.4^o^	2.2^o^
θ*_infinity_	90.0^o^	90.0^o^	90.0^o^
θ*_propeller_	91.7^o^	95.2^o^	25.7^o^
*K*_*T*inf_/*K*_*T*prop_	−1.0%	−0.3%	1.3%
*K*_*Q*inf_/*K*_*Q*prop_	−0.7%	−0.3%	1.1%

For the hull-shaft case, Table 5 compares the results using correction factors evaluated during the initial phase only once at 10° with those evaluated twice at 0° and 10°. In both cases, the correction factors at the effective angles of inclination (around α = 2.2° θ* = 25.7°) are calculated from interpolation in α and from rotation in θ* as explained in Sect. 2.1. Two-point interpolation (α = 10° and −10°) is used in the former case, and three points interpolation (α = 10°, 0° and −10°) in the latter case. Even though the effective direction is closer to 0° than to 10°, the two-point interpolation is enough to provide results with similar accuracy to the three-point interpolation.

**Table 5:** Comparing results from one evaluation of CF (two interpolation points) to those from two evaluations (three interpolation points) for 10° yaw

Hull–shaft Combination	2-point interpolation	3-point interpolation
α_infinity_	10.0^o^	10.0^o^
α_propeller_	2.3^o^	2.2^o^
θ*_infinity_	90.0^o^	90.0^o^
θ*_propeller_	26.0^o^	25.7^o^
*K*_*T*inf_/*K*_*T*prop_	1.0%	1.3%
*K*_*Q*inf_/*K*_*Q*prop_	0.9%	1.1%

The effect of the correction factors on the in-plane forces is shown in Table 6 for the propeller-shaft case at an extreme inclination angle of 30° and for the tractor pod at an angle of 8°. The three components of the forces are given together with the modulus for the sake of completeness. The forces are made non-dimensional using the modulus of the force for the nominal direction of the effective inflow as defined at the end of Sect. 2.

**Table 6:** Comparison of force components and magnitude for computations without (no CF) and with correction factors using a nominal (CF nominal) and global effective (CF effective) direction of evaluation

Case of inclined shaft at 30° angle			
	No CF	CF nominal	CF effective
*Fx/Fc*	0.965	0.913	0.922
*Fy/Fc*	0.402	0.402	0.406
*Fz/Fc*	0.154	0.072	0.076
*F/Fc*	1.057	1.000	1.010

Case of tractor pod at 8° angle			
	no CF	CF nominal	CF effective
*Fx/Fc*	0.929	1.000	1.003
*Fy/Fc*	0.038	0.022	0.022
*Fz/Fc*	0.030	0.013	0.007
*F/Fc*	0.930	1.000	1.003

The effect of using correction factors is significant for the *x-* and *z-*components of the force. The impact on the *z*-force is especially strong for the case with a large angle of inclination. However, the direction of evaluation in the correction factors, either effective or nominal, affects less the force components.

## Discussion

Three examples are provided on how the CF approach works for controlling RANS-potential flow coupling errors. Concerning the first case, a uniform flow at an incidence angle to a cylindrical shaft is expected to yield a low-speed flow zone over the shaft on the side of the incoming flow, and conversely a high-speed zone on the opposite side. However, when a propeller is rotating fixed to the shaft, the axial circulation induced by the propeller on the flow tends to shift circumferentially the location of such zones at the propeller plane. The angular shift occurs in a way similar to the shift on the stagnation points produced by a uniform flow incident to a cylinder with a longitudinal vortex at its central axis. This effect is a part of the effective wake field of a shaft-propeller configuration at an angle of incidence.

The proposed approach for calculating the effective wake is able to disclose such an effect on the effective field. After subtracting the propeller induced velocities from the total flow at the propeller plane, the resulting effective wake due to the shaft exhibits the locations of axial velocity flow peaks shifted circumferentially on opposite angular directions, approaching each other. Moreover, the locations of the tangential velocity peaks at  ± 90° shift angle are either expanded or contracted similarly to the situation in the idealized cylinder-vortex flow.

For this particular application, the tangential flow is the main contributor for the force fluctuation, and therefore we can expect only a moderate contribution of the axial velocity field on forces. However, in other applications like that of the tractor pod unit at a yaw angle the impact of the axial velocity field would be larger.

Note also that the uncorrected approach tends to increase the circumferential variation of velocity on the propeller disk and consequently, of blade force amplitude, introducing numerical ‘noise’ in the solution. This would lead to a situation similar to that mentioned in the introduction, where coupling errors may obscure the benefits of introducing high fidelity models for propeller simulation.

Focusing now on the podded propulsor case, it is clear from the results that the effect of the strut and fin on the effective flow field is larger than that of the single shaft for ordinary maneuvering yaw angles around 10°. The direction of the propeller rotation together with the lack of vertical symmetry caused by the different shape of the strut and the fin contributed to the lack of a fully symmetrical port-starboard solution with the yaw angle. The effect of the correction factors on the performance coefficients is significant.

For the hull case with a single propeller shaft, the influence of the correction factors is found to be more significant on the propeller performance coefficients than on the resistance coefficient of the hull. The asymmetric behaviour of the keel vortex on the effective wake has been illustrated. Depending on the sign of the yaw angle (or alternatively, depending on the sign of the propeller rotation), the keel vortex will be either attracted to or repelled from the propeller disk with a clear impact on the propeller effective wake.

The similar results obtained with correction factors interpolated from two or three interpolation points (corresponding to one or two CF evaluations in the initial phase, respectively) is indicative of the predictable CF behaviour at 0°, which can be obtained from CF interpolation between positive and negative incidence α angles. This reduces in most practical situations the number of CF evaluations in the initial phase to one, that of the maximum oblique angle expected.

Concerning the thickness of the region where the body forces are distributed in the axial direction, we recommend keep things simple and even use one-cell thickness layer. Once the correction factor approach is used, the error in the propeller induced velocity and effective wake distribution linked to the chosen interface thickness is controlled by the correction factor approach. This makes irrelevant the choice of the interface thickness in most practical cases.

The sensitivity of the correction factors to the in-plane loads has been illustrated for an inclined shaft-propeller configuration at an extreme inclination angle of 30° and for the tractor pod at an angle of 8°. As it would be expected, the in-plane loads are affected by the correction factors. In these particular cases, the components perpendicular to the direction of inclination (*F*_*z*_) are more affected than those on the inclination direction (*F*_*y*_). For special propellers like those with tip-modified geometries (CLT, tip loaded…), potential flow solvers with a fidelity level of at least lifting-surface precision should be used so that not only the circumferential but also the radial in-plane loads are properly evaluated.

A final remark is that the correction factors depend on the nature of the potential/viscous flow solvers, their coupling and implementation into a computational grid. In principle, they should not be indiscriminately transferred between different types of solvers. Each coupled RANS/potential flow method has its own modelling errors. Different solvers require different corrections to obtain the final solution of the effective wake.

The effective flow calculated with the proposed approach is more “code-independent” than that calculated with current approaches since we get rid of non-physical coupling errors. Such independency from the propeller solver can be considered from two points of view. On the one hand, the effective wake includes no propeller-induced velocities by definition, (propeller induced velocities are removed from the total velocities). On the other hand, coupling interaction errors in the induced velocities are also removed. This makes it possible also to use a simple and fast potential flow code like lifting line during the convergence iterations and a more sophisticated one like panel method after convergence for detailed analysis.

## Conclusion

The accuracy of predicted forces in current numerical methods that couple potential and viscous flow models deteriorates for oblique inflows, especially at high loadings where coupling errors are larger. The proposed CF approach is expected to improve numerical simulations for propeller design and analysis, including maneuvering, by reducing coupling errors and allowing the use of faster potential flow models for the propulsor.
